# Systematic Literature Review of the Nutrient Status, Intake, and Diet Quality of Chinese Children across Different Age Groups

**DOI:** 10.3390/nu15061536

**Published:** 2023-03-22

**Authors:** Katie Ayling, Rongrong Li, Leilani Muhardi, Alida Melse-Boonstra, Ye Sun, Wei Chen, Urszula Kudla

**Affiliations:** 1 Friesland Campina, 3818 LA Amersfoort, The Netherlands; katie.ayling@frieslandcampina.com; 2 Department of Clinical Nutrition, Peking Union Medical College Hospital (PUMCH), Beijing 100730, China; 3 Friesland Campina AMEA, Singapore 039190, Singapore; 4 Division of Human Nutrition and Health, Wageningen University & Research, 6703 HE Wageningen, The Netherlands; 5 Friesland Campina Development Centre AMEA, Singapore 118261, Singapore

**Keywords:** children 0–18 years of age, China, nutrient status, nutrient intake, diet quality

## Abstract

There is a lack of comprehensive reports on nutrient deficiencies and dietary intake among various age groups of children in China. The objective of this review is to provide an overview of the nutrient status, intake, and diet adequacy of Chinese children (0–18 years old). PubMed and Scopus were searched for literature published between January 2010 and July 2022. A systematic review approach with a quality assessment was performed to analyze 2986 identified articles in English and Chinese. Eighty-three articles were included in the analysis. In younger children, anemia and iron and Vitamin A deficiencies remain severe public health problems, despite high Vitamin A and adequate iron intake. In older children, a high prevalence of selenium; Vitamin A and D deficiencies; and inadequate intakes of Vitamins A, D, B, C, selenium, and calcium were reported. Intakes of dairy, soybeans, fruits, and vegetables were below recommended levels. High intakes of iodine, total and saturated fat, and sodium and low dietary diversity scores were also reported. As nutritional concerns vary with age and region, future nutrition interventions should be tailored accordingly.

## 1. Introduction

Malnutrition can have a significant and often irreversible adverse impact on a child’s survival and growth, their ability to learn in school, and productivity in later life [1]. Failure to provide optimal nutrition can lead to malnutrition in the long term. The different forms of malnutrition are often referred to as a triple burden: undernutrition (underweight, stunting, and wasting), vitamin or mineral deficiencies, and overnutrition (overweight and obesity), resulting in diet-related non-communicable diseases [2].

China is the most populous country in the world, with approximately 1.4 billion people and one-fifth of the total number of children [3]. Recent decades of rapid socioeconomic development, urbanization, and modernization in China have greatly improved children’s nutritional status [4]. There was a significant reduction in undernutrition and related mortality. China is one of the few countries on course to meet the nutrition-related targets for stunting and wasting under the Sustainable Development Goals (SDG) of the United Nations, and some progress has been made for low birth weight [5,6]. The rates of stunting were reduced by 70% (from 33.4% in 1990 to 9.9% in 2010) and underweight by 84% (from 19.1% to 3.6%) among Chinese children under 5 years. Similarly, stunting and underweight decreased in Chinese children and adolescents (7–12 years) from 2.4% in 2009 to 0.4% in 2011, and 24.6% in 2009 and 21.4% in 2011 [7,8]. 

In contrast, China has made no progress towards the SDG targets on the prevalence of overweight; instead, there has been a transition with the proportion of overweight children now exceeding the prevalence of undernutrition indicators. From 2000 to 2016, the rate of overweight and obesity among children 1–4 years increased from 9.5% and 3.9% to 11.9% and 6.9%, respectively [9]. In children and adolescents (7–12 years), the rate of overweight and obesity also increased, from 11.3% and 2.9% in 2009 to 12.6% and 6.3% in 2011 [8]. In slightly older children and adolescents (6–17 years), the rate of overweight and obesity increased from 9.0% and 7.2% in 2009 to 11.5% and 10.5% in 2011. The most recent estimates in 2015 showed further increases of 11% and 12.7%, respectively [10].

Information on the nutritional status (e.g., stunting) of Chinese children is publicly available in reports by various organizations such as the United Nations Network on nutrition (UNICEF, WHO, and FAO). Most of the data are derived from the China CDC (Chinese Control and Prevention) and are based on its 2011–2013 and 2015–2017 China Nutrition and Health Surveillance. However, a comprehensive report on nutrient deficiencies and dietary inadequacies across various age groups and geographical areas is not as readily available. It is hypothesized that due to the size of the country and rapid economic transition, there would be geographical differences across regions. Therefore, apart from national studies, we have also included an overview of subnational studies, as they frequently provide more in-depth information for more limited geographical areas. The objective of this paper is to provide an overview of the nutrient status, intake, and diet adequacy of Chinese children (0–18 years old) across various age groups and regions using a systematic review approach.

## 2. Materials and Methods

### 2.1. Guidelines and Registration

**Figure 1:**
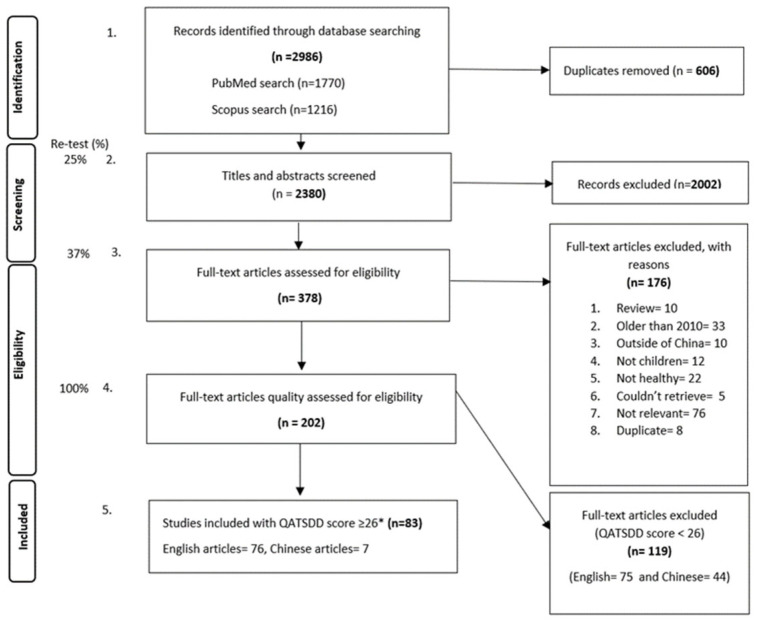
PRISMA flow chart. * QATSDD score cut off reflected the third tertile for all the retrieved articles. Special weightage was given to criteria 5, ‘Representative sample of target group of a reasonable size’, whereby all observational studies with a maximum score (3) were included in the review regardless of the total QATSDD score.

This systematic review was conducted following PRISMA guidelines (Figure 1), with the study protocol registered under PROSPERO International Prospective Register of Systematic Reviews (registration number: CRD42020220110).

### 2.2. Data Sources and Study Selection

Scopus and PubMed were searched for articles published in English and Chinese between January 2010 and July 2022 providing information about ‘nutrient’ and ‘diet’, ‘status’, ‘intake’, and ‘inadequacy’ of healthy ‘Chinese’ ’children’ aged 0–18 years old. The relevant literature was searched using the above keywords/terminology and following the PICOS (population, intervention, comparison, outcome setting/design) approach, as displayed in Appendix A (Table A1). Furthermore, the reference lists of existing reviews and previously identified articles were examined individually to complement the electronic search.

### 2.3. Data Extraction

Four authors (UK, KA, LM, and RL) independently screened titles/abstracts and full-text articles for eligibility against a priori defined inclusion/exclusion criteria. After removing duplicates, the relevant studies were grouped into 4 different categories, namely, Chinese papers, nutrient status, nutrient intake, and food intake/diet quality. 

### 2.4. Quality and Bias Assessment

Each of the four authors assessed the quality and bias of the included studies in one topic classification based on the ‘Quality Assessment Tool for Reviewing Studies with Diverse Design’ (QATSDD) [11]. These reviewers independently awarded each included paper quality scores by assessing each QATSDD criterion (for example, ‘Description of the procedure for data collection’) on a 4-point scale from 0 to 3 (0 = the criterion is not at all described, 1 = described to some extent, 2 = moderately described, and 3 = described in full). The resulting scores were then expressed as a percentage of the maximum possible score of 42. The sum of the scores for all relevant QATSDD criteria reflected the overall quality of each paper. The reviewing team decided to only include articles with a cutoff score of 26 or higher, which reflected the third tertile of the QATSDD score for all the retrieved articles. Special weightage was given to criteria 5, ‘Representative sample of target group of a reasonable size’, whereby all observational studies with a maximum score (3) were included in the review regardless of the total QATSDD score. This allowed all studies with large and representative sample sizes to be included in the review regardless of scoring on other quality criteria. The QATSDD assessment was then repeated by a different reviewer. In the case where the difference in scoring between reviewer #1 and reviewer #2 was larger than 4 points, a third reviewer further assessed the article.

### 2.5. Data Analysis

Data were then extracted and tabulated into standardized tables for nutrient status, nutrient intake, and food group intake (which included diet quality scores); outcome indicators are summarized in Table 1. A second reviewer repeated the data extraction on 5% of all full texts for quality purposes. For continuous outcomes with 3 or more data points (hemoglobin status, Vitamin A intake, and Vitamin D status), mean and standard errors (SEs) of the levels/intakes were pooled by meta-analysis and visualized using a forest plot in Stata version 17, 2021 (StataCorp LLC, College Station, TX, USA). For outcomes with different cutoffs across age groups (hemoglobin status and Vitamin A intake), pooled estimates were calculated within each age group. Subgroups other than age groups within the same study were combined to create a single study point for simplicity. If SEs were not reported, they were calculated or estimated from SDs, CIs, or pooled from studies reporting sufficient information. Inverse variance weighting was used to pool the different studies/publications. Results from the random-effects model were presented, considering the large between-study heterogeneity (quantified by the I2 statistic) in most cases.

Where 3 or fewer data points were available per age group, the data points were tabulated per nutrient or food group. The prevalence of nutrient deficiencies, insufficiencies, and inadequacies were reported based on cutoff levels used in the original articles (Table 1). Nutrient deficiencies refer to the supply of nutrients below requirements that may manifest as clinical symptoms. Nutrient insufficiencies are the intermediate situation and may be associated with subclinical functional deficits and/or enhanced risks of pathologies. Nutrient inadequacies refer to nutrient intakes below the recommended amount [12]. Mean nutrient intake values were reported and classified against the Chinese estimated average requirement (EAR) and Upper Limit (UL) [13]. Food intake was reported in g/day and % below recommendations (based on original article). Food group intake (g/day) was classified as adequate or inadequate based on the recommended daily intake from the Chinese Nutrition Society’s Food Guide Pagoda [14]. Diet Quality was measured by scoring food patterns in terms of how closely they aligned with national dietary guidelines and how diverse the variety of choices was. The score was recorded, as was the scoring system used [15].

**Table 1:** Summary of nutrient status, nutrient intake, and food intake outcome indicators.

Category	Outcome Indicator	Biomarker/Assessment Method	Outcome Indicator Measure (Units)	Cutoffs for Deficiency, Insufficiency, or Inadequacy
Nutrient Status	Iron status	Hemoglobin concentration (Hb)	Mean (g/L), SE ^1^	Prevalence of anemia: as per WHO guidelines, age-specific groups: for children <5 years <110 g/L anemia, for children aged 5–11 years <115 g/L anemia, for children aged 12–14 years <120 g/L anemia, for non-pregnant women (15 years and above) <120 g/L anemia, and for men (15 years and above) <130 g/L [16]
		Serum ferritin	Mean (μg/L), SE ^1^	% Iron deficiency: as per WHO guidelines, <12 μg/L for non-infected children or <30 μg/L for infected children (%, *n*) [17]
	Vitamin A status	Serum retinol concentration	Mean (μmol/L), SE ^1^	Vitamin A deficient (VAD): as per WHO guidelines, 0.7 μmol/L (<20 µg/dL), % Vitamin A Insufficient (VAI): 1.05 μmol/L (<30 µg/dL) [18]
	Vitamin D status	25(OH)D concentration	Mean (nmol/L), SE ^1^	Vitamin D deficiency (VAD): the clinical practice guidelines of the Endocrine Society Task Force on Vitamin D have defined a cutoff level of <20 ng/mL (50 nmol/L) as Vitamin D deficient, % Vitamin D insufficient: levels between 20 and 30 ng/mL (50–75 nmol/L) as ‘insufficient’ [19]. More recent recommendations justify a cutoff of 12 ng/mL (30 nmol/L) for deficiency
	Iodine status	Urinary iodine Presence of goiter	Median (ng/mL), SE ^1^	% Iodine deficient: as per WHO guidelines, <100 μg/L as deficient, 100–300 μg/L as sufficient, and >300 μg/L as excess [20] % children with goiter
	Zinc status	Serum zinc concentration	Mean (μg/L), SE ^1^	% Zinc deficient: <700 μg/L as deficient [21]
	Selenium status	Serum selenium concentration	Mean (μg/L), SE ^1^	% Selenium deficient: <45 μg/L as very low, % Low selenium: <60 μg/L as low and 60–120 μg/L as normal [22]
	Vitamin B12 status	Plasma Vitamin B12	Mean (pmol/L), SE ^1^	% Vitamin B12 deficient: as per WHO guidelines: <150 pmol/L (203 pg/mL) [23,24]
	Folate status	Serum/plasma folate level	Mean (nmol/L), SE ^1^	% Folate deficient: as per WHO guidelines, 4 ng/mL (<10 nmol/L) as deficient [23]
Nutrient (and Energy) Intake	Energy intake	Self-reported from 24 h recalls (24 h). In most cases, multiple 24 h	Mean (Kcal/day), SE ^1^	EER cutoff method using Chinese estimated energy requirement (EER) [13]
	Macronutrients (carbohydrates, fat, and protein) and micronutrients (iron; zinc; selenium; calcium; iodine; sodium; Vitamins A, B1, B2, B3, B9, B12, D)	Self-reported from 24 h recalls (24 h). In most cases, multiple 24 h	Mean (g mg, μg)/day, SE ^1^	% inadequacy: % population that consumed below the EAR. Calculated using the EAR cutoff method using Chinese-specific estimated average requirement (EAR) and Upper Limit (UL) [13]. Adequate Intakes (AI) were used when EAR was not possible
Food Intake	Food group intake	Self-reported from 24 h recalls (24 h). In most cases, multiple 24 h	Mean (g/day)	Food group inadequacy; % below recommended; recommendations based on the Chinese Nutrition Society’s Food Guide Pagoda [14]
Diet Quality	Diet quality	Assessment method varies by study; see Appendix B	Diet diversity score	Scoring system varies by study; see Appendix B

^1^ If SEs (standard errors) were not reported, they were calculated or estimated from SDs, CIs, or pooled from studies reporting sufficient information.

## 3. Results

### 3.1. Study Characteristics

A total of 202 eligible articles were identified from the literature search, of which 83 met the inclusion criteria for further analysis. Twenty-five studies (30%) were based on national surveys. However, these national studies do not cover all provinces in China. The remaining publications (*n* = 58, 70%) were subnational studies, mostly from western China (*n* = 28). Almost half of the studies (47%) included children from the general population (both rural and urban or unspecified), and around 47 studies (57%) included nutrient status indicators (Table 2). 

### 3.2. Energy and Macronutrient Intake

#### 3.2.1. Energy Intake

The average energy intake for infants (0–6 months), older children (7–12 years), and teenagers (13–18 years) was reported to be below the recommended EER threshold [29,38,39,42,63,97]. Older infants (from 6 months on) and younger children (1 to 6 years old), were, on average, meeting their energy requirements [38,42,62,77,84] (Table 3).

#### 3.2.2. Fat Intake

Fat intake in children (0–17 years) was reported between 24.7 and 66.2 g/day [29,62,63,97] and 35.8 and 37.5% of energy intake (EI) [29,38]. Subnational studies reported that children’s mean fat intake (%EI) was below the adequate intake (AI) for children aged 0–3 years old. National data for children aged 4 to 17 years reported that fat intake (%EI) was above the recommended intake (Table 3). 

#### 3.2.3. Carbohydrate Intake

CHNS 2011-12 and 2015 reported that the contribution of carbohydrates to energy intake for children between 12–17 and 6–17 years was 59% and 51%, respectively; this was within the recommended 50–65% [29]. The mean carbohydrate intake (%EI) of those living in provinces was 55%, megacities 47%, females 57.7%, and males 59.9% of energy intake [39]. Subnational studies covering three regions of China (east, central, and west) reported that children 0–10 years met the EAR for mean carbohydrate intake (Supplementary Material Table S1) [62,77,84,97]. CHNS 2011-12 reported total and added sugar at 26 g and 9 g/day for children between 4 and 13 years old [37]. 

#### 3.2.4. Protein Intake

CHNS 2011-12 data reported that children 12–17 years consumed 12.9% of their energy intake from protein, and this was within the recommended 10–30% [39,110]. CHNS 2015 and subnational studies covering all three regions (east, west, and central China) reported protein intakes above the EAR for all age groups (Supplementary Material Table S2) [29,50,51,62,63,77,84,96,97].

### 3.3. Mineral Intake and Status 

#### 3.3.1. Anemia and Iron Status

**Figure 2:**
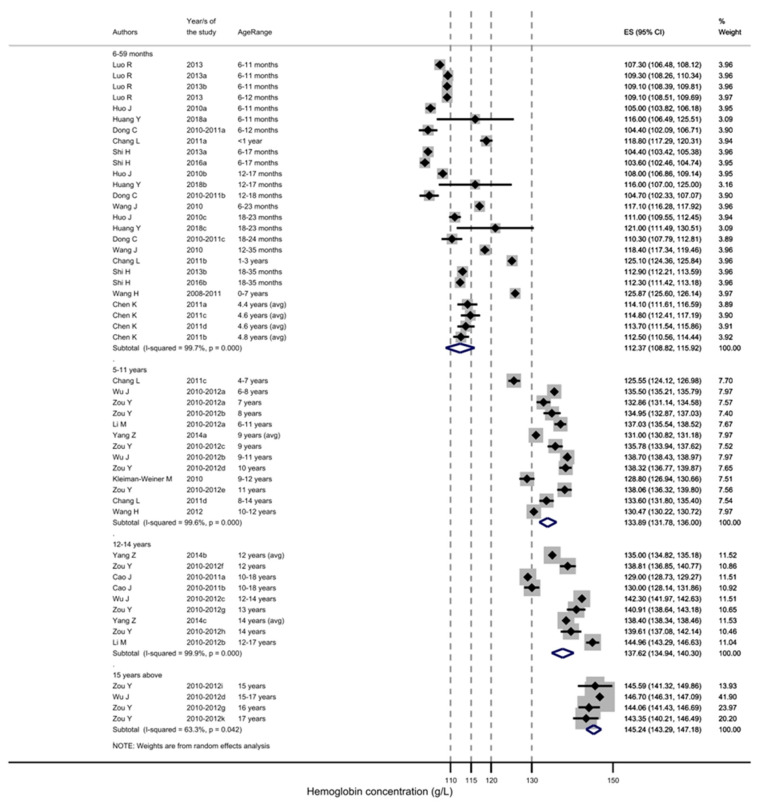
Hemoglobin concentration (g/L) per age group of Chinese children [30,31,47,52,55,57,61,67,80,97,102,103,105]. Dotted gray line represents WHO-indicated hemoglobin concentration to diagnose anemia at sea level (g/L). For children 15 years and above, separate concentrations for females and males are given.

Anemia prevalence in 0–18-year-old children from China in national [30,31,47] and subnational studies (mostly covering the western region) ranged from 2.7 to 86%, and ID prevalence ranged from 7.3% to 62% [50,52,55,56,57,60,61,64,65,67,68,75,87,95,97,102,103,105]. The prevalence of anemia in children decreased with age (Table 4). The average hemoglobin concentrations across different age groups were significantly different in the Huang Y et al., 2019 study in western China (Figure 2) [55]. 

The overall prevalence of anemia in rural settings ranged from 5.6 to 86%, and that in urban settings between 2.7% and 17%. Anemia prevalence in children aged 0–23 months in rural settings ranged from 17 to 86% and that in urban settings was between 6.3 and 17%, whereas children at 24–35 months in rural settings ranged from 5.6 to 42% and those in urban settings between 4.5 and 20% [52,53,55,56,57,61,64,65,67,68,75,87,102,103,105]. In rural children aged 9, 12, and 14 years, the mean hemoglobin level was significantly lower (134.7 ± 12.6 g/L vs. 135.6 ± 13.0 g/L, *p* < 0.001) and the prevalence of anemia was significantly higher (8.9% vs. 8.0%, *p* < 0.01) compared with their urban peers. When analyzed stratified by sex and age, the difference between rural and urban anemia prevalence remained significant only in 12- and 14-year-old girls [47].

Similarly reported in the Nutrition Study of Pre-school Children and School Children, for children 3–11 years in seven cities and two villages across east, west, and central China, the prevalence of iron deficiency (ID), as measured by serum ferritin levels, significantly decreased with age (*p* < 0.001) [82]. 

#### 3.3.2. Iron Intake

The iron intake of 0–18-year-old children in China from national and sub-national studies (covering west and east China) ranged between 3.1 and 30 mg/day [28,36,42,50,51,62,84,96,97]. Inadequate intake was reported for children (3–17 years) in national studies between 10 and 24% [28,36,42]. CHNS 2015 reported <10% of normal-weight boys (6–17 years) and approximately 20% of normal-weight girls (6–12 years) were inadequate in iron [28]. Younger children met or were close to meeting EAR iron intake levels (Table 4). 

#### 3.3.3. Zinc Status 

The prevalence of zinc deficiency in children (6–11 years) from CHNS 2011-12 was reported at 10% [35]. A recent study by Tian et al., 2020, reported zinc deficiency in children (7–17 years) at 4.8% in Jiangsu province, eastern China [95]. 

#### 3.3.4. Zinc Intake

Zinc inadequacy appears to increase with age. Data from CHNS 2011-12 showed children between 11 and 17 years had the highest prevalence of inadequacy, between 38 and 42%, followed by children aged 7–10 years at 28% and children 4–6 years at 24% [36]. CHNS 2015 reported that approximately 30% of children (6–17-year-old normal-weight children) had zinc inadequacy [28]. The MING study showed children between 0 and 6 months had a zinc inadequacy between 0.4 and 19% based on the type of feeding (breast and artificial, respectively) [63]. Further sub-national data are given in Table 4 [51,77,96,97]. 

#### 3.3.5. Selenium Status 

CHNS 2011-12 reported that selenium deficiency (<60 μg/L serum selenium concentration) prevalence in children (6–11 years) was 44% and very low status (<45 μg/L serum selenium concentration) was 25%, with a median serum selenium concentration of 74.2 μg/L [22]. A lower prevalence of deficiency was reported in a middle school (children aged 12–14 years) in Xi’an, Shaanxi, central China at 3.1–3.6% [107]. No significant difference in serum selenium concentration was observed between sexes or age groups [22].

#### 3.3.6. Selenium Intake

Two studies reported selenium intake data from CHNS 2011-12. Lui et al., 2017 reported that children 6–11 years old had an overall median dietary selenium intake of 33.6 μg/day, and the prevalence of selenium inadequacy was 52% [22]. Wang et al., 2017 reported that for children 4–17 years, inadequacy ranged between 50.8 and 71.8%, which appeared to increase with age (Table 4) [36]. Hu et al., 2021 reported CHNS 2015 data showing approximately 65% selenium inadequacy in normal-weight boys and 70% in normal-weight girls (6–17 years) [28]. Tian et al. reported selenium intake of 70 μg/day (above the EAR (25–45 μg/day)) among 3–5-year-old children living in rural Hubei province. This, in combination with the selenium level in drinking water of 0.04μg/L, was suggested as an increased risk of toxicity [79], but not yet to the level of the UL between 100 and 150 μg/day. 

#### 3.3.7. Calcium Intake 

Calcium intake of 0–17-year-old children in national [28,36,42] and sub-national studies across west and east China [50,51,62,63,84,96,97] was between 236 and 801 mg/day, with 92–96% inadequacy in intake; this appeared to increase with age. CHNS 2011-12 and CHNS 2015 data reported a high prevalence of calcium inadequacy in children 4–17 years [28,36]. In younger children (0–11 months), the mean calcium intake was above the AI. For children 1–3 years old, sub-national studies differed, with one study below the EAR [51], one above the EAR [50], and the other above both the EAR [62]. The mean intake of calcium in older children (4–17 years) did not meet the EAR [36,77,84,96,97]. 

#### 3.3.8. Magnesium Intake

Magnesium intake in children (6 months–17 years) was reported in national studies, CHNS 2011-12 and CHNS 2015, and sub-national studies, between 113 and 2798 mg/day [28,42,62,77,84]. The CHNS 2015 study showed a rate between 50 and 60% magnesium inadequacy in normal-weight boys and girls (6–17 years) [28]. For all age groups, the mean magnesium intake was above the EAR, except for those 11 to 13 years.

#### 3.3.9. Sodium Intake

Sodium intake in children (0–17 years) was reported to be between 67 and 6100 mg/day [36,39,42,62,63,77,97]. The prevalence of inadequacy was not reported for any age group. The mean sodium intake of younger children (0–11 months) was below the AI, and children from 1 to 17 years had a mean sodium intake above the AI and proposed intake for preventing non-communicable disease (PI-NCD) (Table 4) [39,42,63,77].

#### 3.3.10. Iodine Status

Iodine deficiency prevalence, as measured by the presence of goiter in children (8–10 years), ranged from 2.2 to 14%, with 2.2% being the national average and 11–14% in Hebei province [46,100,106]. The median urinary iodine concentration (UIC) nationally for children (8–10 years) was less than 250 μg (227–239 μg/L nationally in 2011; 211 μg/L in Hebei, Guangxi and Zhejiang; and 191 μg/L in Zhejiang province) [45,46,86,100,106]. In Hebei, the median UIC was 419 μg/L; additionally, 68% of the study populations were > 300 μg/L, while this was 28% at the national level [46,100]. Two studies in Zhejiang province showed higher UIC in rural populations versus urban; however, this was not significant [86,101]. Pre-schoolers (3–6 years) had a median UIC of 181 μg/L for those living in rural areas and 182 μg/L for those living in urban areas; school-aged children (7–12 years) had a median of 191 μg/L for those living in rural areas and 166 μg/L for those living in urban areas; and adolescents (13–18 years old) had a median of 193 μg/L for those living in rural areas and 186 μg/L for those living in urban areas [86]. Similarly, in another study, children aged 8 years had UIC of 158 μg/l in rural areas and 161 μg/L in urban areas; children aged 9 years had 180 μg/L in rural areas and 157 μg/L in urban areas; and children aged 10 years had 185.4 μg/L in rural areas and 180 μg/L in urban areas [101]. 

No iodine intake data for children were found.

#### 3.3.11. Phosphorus Intake

For all age groups, the mean phosphorus intake was above the EAR; refer to Supplementary Material Table S3 [42,62,63,77,97]. CHNS 2015 showed approximately 10% phosphorus inadequacy in normal-weight boys and girls (6–17 years) [28]. 

### 3.4. Vitamin intake and Status 

#### 3.4.1. Vitamin A Status 

The prevalence of Vitamin A deficiency (VAD) in children (0–17 years) ranged from 0 to 27% and Vitamin A insufficiency (VAI) from 15 to 58% [32,40,41,69,78,81,95,105]. A total of four studies reported VAD using CHNS 2011-12 data: for children 6–12 years, VAD was 6.6–8.0% (VAI 17–23%), and for children 12–17 years, it was 4.5–7.2% (VAI 14–21%) [33,40,41].

Yang C et al. used a higher cutoff of <30 µg/dL serum retinol concentration (same as VAI) than was used in other studies based on WHO criteria (<20 µg/dL serum retinol concentration), and reported a higher prevalence at 27% for children 7–17 years [32].

VAI appeared to peak in the 1–3-year-old group, between 43.3 and 54.1% [67,68,74], and to go down with age to approximately 14.32–21.13% (Table 5) [32,40,41,95,105]. One study by Wang et al. in central China reported a higher prevalence of VAD, namely 20% (VAI 41%) in children 0–12 years, compared with the CHNS studies mentioned above and other sub-national studies in western China [67,68,80,81,105]. In this study, VAD decreased with age; however, significance was not assessed, and no difference between sexes was found [74].

CHNS 2011–2012 data reported VAD among 6–17-year-old children. The reported prevalence of VAD was not significantly different between children in large cities (8.0%) vs. small- and medium-sized cities (7.6%) and between age groups for 6–11-year-old children (8.0%) vs. 12–17-year-old children (7.2%) [40]. The prevalence of VAI among children in large cities was 19% vs. small- and medium-sized cities (19%) and did not significantly differ. However, a significant difference was seen between 6–11-year-old children (17%) and 12–17-year-old children (21%) [40,41]. The prevalence of VAD in urban vs. rural Chinese children was also determined. However, no test of difference was performed. Children aged 6–9 years living in urban regions had a VAD of 10.7%, and those living in rural regions had a VAD of 8.9%. Children aged 10–13 years living in urban regions had a VAD prevalence of 7.6%, and those living in rural regions had a VAD prevalence of 5.7% [33]. A significant difference in VAD prevalence was reported for children of 6–17 years living in towns without high poverty levels (6.1%) vs. towns with high levels of poverty (4.6%) and for 6–11-year-old children (6.6%) vs. 12–17-year-old children (4.5%). The prevalence of VAI for children in towns without high levels of poverty was 19%, whereas this was 18% for children in towns with high poverty levels and 23% for 6–11-year-old children vs. 14% for 12–17-year-old children [41].

#### 3.4.2. Vitamin A Intake

**Figure 3:**
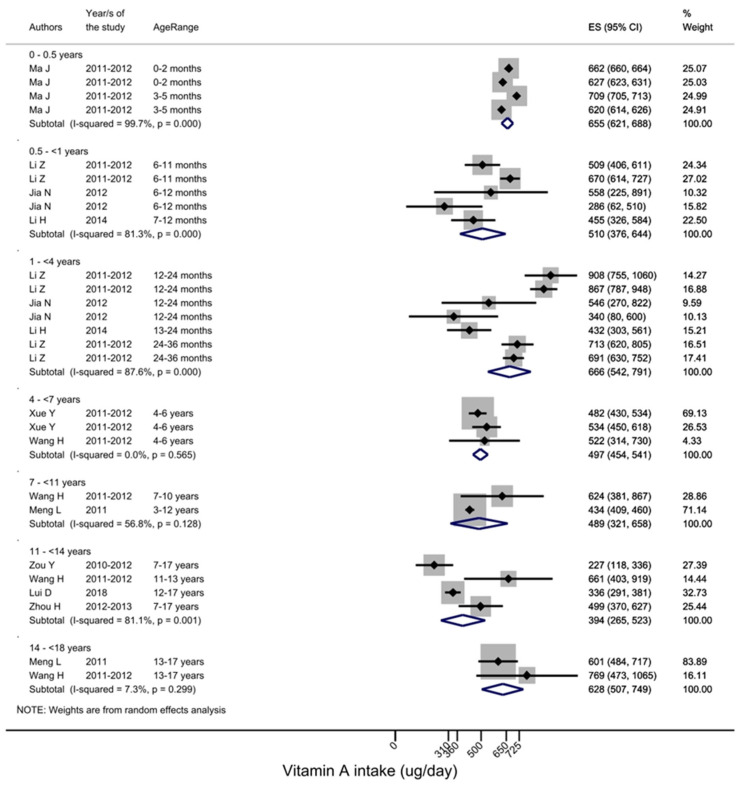
Vitamin A intake across different age groups [36,42,50,51,62,63,84,96,98,108]. EAR cur-off points are age-specific, for 1–3-year-olds 220 μg RE/day, 4–6-year-olds 260 μg RE/day, 7–10-years-old 360 μg RE/day, 11–13-years-old 480 μg RE/day for males and 250 μg RE/day for females, 14–17-years-old 590 μg RE/day for males and 450 μg RE/day for females.

Vitamin A intake in Chinese children (0–18 years) ranged from 223 to 769 μg RE/day, and inadequate intake ranged between 0.4 and 70% (Table 5 and Figure 3) [28,36,42,63,77].

#### 3.4.3. Vitamin B1 (Thiamin) Intake

Vitamin B1 intake for children (0–18 years) was between 0.2 and 1.2 mg/day [36,42,50,62,63,84,97], with an inadequate intake prevalence for children over 4 years old ranging between 68 and 92% [28,36,42]. The prevalence of inadequate Vitamin B1 intake increased with age (Table 5). For younger age groups, no prevalence levels were reported; however, the mean Vitamin B1 intake was higher than the AI for children 0–11 months. Between 1 and 3 years, the mean intake was below the EAR and RNI [50,62,77]. Vitamin B1 status information was not available. 

#### 3.4.4. Vitamin B2 (Riboflavin) Intake

Vitamin B2 intake for children (0–18 years) was between 0.5 and 1.7 mg/day [28,36,42,50,62,63,84,97], with an inadequate intake prevalence for children over 4 years old ranging between 66 and 86% [28,36,42]. The prevalence of Vitamin B2 inadequacy increased with age, as reported in the CHNS 2011-12 [36] (Table 5). The prevalence of inadequacy was not reported for younger children (0–35 months). The lowest Vitamin B2 intake was reported in children 4–17 years in Zhejiang province, and the highest was reported in the national MING study for children 12–23 months of age in eight cities (Beijing, Guangzhou, Shanghai, Chengdu, Lanzhou, Shenyang, Zhengzhou, and Suzhou) [62,97]. Vitamin B2 status information was not available. 

#### 3.4.5. Vitamin B3 (Niacin) Intake

Vitamin B3 intake for children (0–17 years) was between 2.1 and 15.9 mg/day [42,50,62,63,84,97] (Table 5). The mean Vitamin B3 intake was above the AI and EAR for children 0–10 years old. The mean intake of Vitamin B3 from national data for children 11–17 years met the EAR; however, some sub-national values did not meet the EAR. 

Vitamin B3 status information was not available. 

#### 3.4.6. Vitamin C Intake

Vitamin C intake for younger age groups (0–6 years) met the AI or EAR, whereas for older groups (7–17 years), mean intake from national and sub-national studies was lower than the EAR [36,42,50,62,63,84,97]. CHNS 2015 that reported approximately 65% of normal-weight children 6–17 years old had Vitamin C inadequacy [28]. The prevalence of Vitamin C inadequacy increased with age, as reported in the CHNS 2011-12 [36] (Table 5). Vitamin C status information was not available. 

#### 3.4.7. Vitamin D Status 

The prevalence of Vitamin D deficiency (VDD) and insufficiency (VDI) in children (0–18 years) ranged from 3.5% in west China, Yunnan [58], to as high as 69% in central China, Harbin [73], with the majority of the studies placing it in the range between 20 and 30% [70,72,74,75,89,90,91,92,95,98]. 

**Figure 4:**
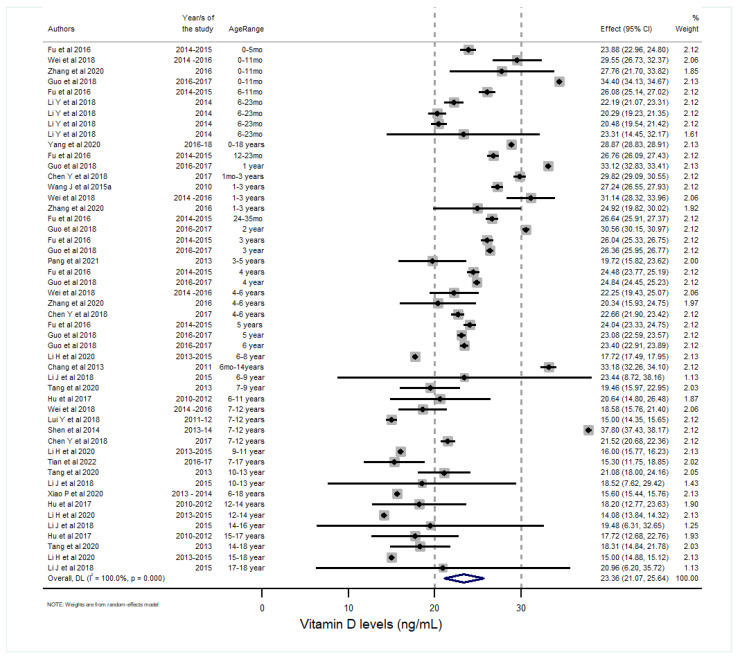
The Vitamin D concentration in Chinese children across different age groups [26,34,58,67,70,71,72,73,74,75,76,85,87,89,89,90,91,99]. The clinical practice guidelines of the Endocrine Society Task Force on Vitamin D have defined a cutoff level of <20 ng/mL (50 nmol/L) as Vitamin D deficient and levels between 20 and 30 ng/mL (50–75 nmol/L) as ‘insufficient’ [19]. More recent recommendations justify a cutoff of 12 ng/mL (30 nmol/L) for deficiency.

The decreasing Vitamin D concentration and increasing VDD with age are shown in Figure 4 [19,26,71,76,99] and Table 5. National data from CHNS 2011-12 for children 6 to 17 years old reported VDD at 53% (48.2 (35.4–63.4) nmol/L) [34]. The Nutrition Study of Pre-school Children and School Children 2012 for children between 7 and 12 years across 5 cities and 2 villages in East, West, and Central China reported VDD at 55% (15.0 ± 7.9 ng/mL) [85]. 

#### 3.4.8. Vitamin D Intake

Vitamin D intake was reported in children 7–12 years old from The 2012 Nutrition Study of Pre-School children and School Children study at 1.1 µg/day, where the intake of 84% was below the EAR, 86% was below the RNI, and 8.9% was over the UL. The study showed that a higher quartile of Vitamin D intake was associated with a 16% reduction in the risk of Vitamin D deficiency (*p* < 0.001) [85].

#### 3.4.9. Vitamin B12 Status 

There are limited studies on the prevalence of Vitamin B12 deficiency. Two studies located in west China reported that Vitamin B12 deficiency in children 6–35 months living in rural areas was between 0 and 20% [55,67]. In rural Huzhu county, Qinghai province, Vitamin B12 deficiency was reported at 0% in children 6–23 months of age [55], whereas in three counties with high levels of poverty (Wuding, Zhengan, and Zhenan) it was reported at 20% in children 12–35 months of age (347 ± 165 pg/mL) [67]. No dietary intake data were reported.

#### 3.4.10. Folic Acid Status

The folic acid status in children (6–35 months old) was reported between 10.8 and 23 ng/mL, with 13–14% being deficient [55,67] in rural Huzhu County, Qinghai province [55], and three counties in poverty, Wuding, Zhengan, and Zhenan [67].

#### 3.4.11. Folic acid Intake

Folic acid intake was reported in the MING study in eight urban cities of children 6–35 months and was reported to be above the AI and EAR [62]. 

### 3.5. Food Group Intake 

The amount consumed (g/day) per food group and the percentage below recommendations are displayed in Table 6 and are defined across different age groups and geographical areas. Overall, the intake of milk and milk products was inadequate in 96–99% of children (3–8 years), and the mean intake was below the recommended amount (300 g/day) in all age groups [25,27,38,48,93,108]. Seafood intake was below national recommendations for children living in moderately urbanized and rural areas [38]. Intake of red meat and cereals, tubers, and legumes was above national recommendations for all groups, while for eggs, this was only the case for children living in highly and moderately urbanized areas [27,38,93,108]. All remaining (fruits, vegetables, and poultry) were within national recommendations. CHNS 2011-12 reported sugar-sweetened beverage intake at 2.84 ± 5.26 servings per day [44].

### 3.6. Diet Quality

Diet quality was assessed using several diet quality measures (Appendix B, Table A2) [42,43,49,54,56,59,66,83,88,93,94,104]. The minimum acceptable diet (MAD) was not met by 93–95% of children between 6 and 23 months old in rural Qinghai province, Guinan county, and Huzhu county [61], and by 82% in Shanxi and Guizhou provinces in 2013 [66]. In 2018, 46.4% of those aged 6–23 months in rural communities in Guizhou, Henan, Xinjiang, Hubei, and Hebei met the requirements for MAD [49]. 

Among older children, one study recommended adequate diet diversity, and based on national data of children aged 3–17 years, diet diversity was below the recommendation [53]. Another two studies showed a high proportion of the population not meeting recommendations, with only 8.7% of children 6–17 years meeting the ‘Healthy Diet’ Score based on The Chinese Child and Adolescent Cardiovascular Health (CCACH) study, and 20% of 9–15-year-old children meeting the dietary China Dietary Guidelines Index for Youth (CDGI-Y) score [43].

## 4. Discussion

As seen in the retrieved literature in this review, deficiencies of different vitamins and minerals were reported across various age groups. Anemia and iron deficiency remain severe public health problems in children below one year of age, while selenium deficiency was more frequent in older children. Both problems were frequently reported in western China. VAD remained severe for children of all ages based on national data, while VDD appeared to be less of a problem when abundant sunlight was available (in the summer and in south China).

Nutrient intakes in younger children did not seem to always match the nutrient deficiencies mentioned above. For example, Vitamin A intake was above recommended values, iron was adequate, and diet diversity scores were below recommended. Meanwhile, there was a high prevalence of VAD, VDD, and selenium deficiency in older children, matching the reported inadequacies. The intake of additional nutrients: calcium; magnesium; Vitamins B1, B2, and C; and food groups—dairy, soybeans, fruits, and vegetables—was also inadequate. Nevertheless, energy intake was below the recommendation, whereas fat and sodium intake were above the recommendation, suggesting an imbalanced diet.

Discrepancies between urban and rural areas seem to still exist, with some of the nutrient deficiencies such as Vitamin A, iron, and anemia being more prevalent in rural areas and towns with high poverty levels [33,40,41]. Additionally, children in rural areas tend to have less dietary diversity as than those in urban areas [83]. 

Causes of anemia can be multiple, with usually only half of the anemia cases attributable to iron deficiency and the other half to a lack of other hemopoietic nutrients (Vitamin B12, Vitamin A, and folic acid) or iron absorption enhancers (Vitamin C), infectious diseases, and genetic hemoglobin disorders [111]. Despite the limited number of publications on iron deficiency found in this review, the available data suggest that the majority of anemia cases (in one study, as high as 81%) in Chinese children may be due to iron deficiency [55]. Deficiencies in Vitamin B12 and folic acid were generally low and are unlikely to contribute much to the prevalence of anemia, whereas VAD remained high across all age groups and may contribute. 

Globally, children under 5 years of age have the highest frequency of anemia [112]. A recent Lancet analysis in 2020 reported that 56% of preschool-age children (6–59 months) had at least one of the three core micronutrient deficiencies (iron, zinc, and Vitamin A). This was consistent with this review’s finding of a higher prevalence of anemia, iron, and Vitamin A deficiency in younger aged children (no data were available in this review on zinc deficiency for children under 7 years old). The Lancet analysis reported a low prevalence of Vitamin B12 and folate deficiency from a limited number of countries; this was consistent with this review [113]. 

This review found that the highest anemia and ID prevalence were in children under 1 year old, especially from 6 to 12 months. It is recognized in the literature that at this age, during the complementary feeding phase, when foods are introduced to complement milk feeding, there is an increased risk of nutrient deficiencies, especially ID and IDA [114]. The prevalence of anemia in children under 6 months was found to be 46.1% in one study conducted in three national counties with high poverty levels. For the first 6 months of an infant’s life, it is thought that most newborns have sufficient iron stored in their bodies. However, this is dependent on a number of factors, such as the mother having adequate iron stores during pregnancy, the baby being full-term, with normal birthweight, delayed umbilical cord clamping, and sufficient iron [115]. These factors were not given in this article; however, they may play a role in the high prevalence of ID in infants under 6 months found in this review. After 6 months, breastmilk is low in iron and body stores run out; iron-containing foods are important [115]. 

Exclusive breastfeeding was reported between 0.50 and 33.45% in 17 cities in China between 2005 and 2016 [115]. Due to the low number of articles included in this review on infants under 6 months of age, the impact of breastfeeding on the nutrient status of children could not be concluded. One study by MING et al. in 2014 showed the impact of breastfeeding, mixed feeding, and artificial feeding in the first 6 months on nutritional status and nutrient intake. Artificially fed children showed higher disease prevalence and lower Z scores of length-for-age, weight-for-age, and weight-for-length compared with those in breastfed infants. Mostly adequate nutrient intakes were found, except for Vitamin A and zinc, where many artificially fed infants were above the Upper Limit [63].

The prevalence of anemia in Chinese children was positively associated with anemia in mothers [67] and with monotonous diets [55], and was negatively associated with the consumption of meat [55], egg, and milk [47]. The consumption of animal-sourced foods, red meat, and eggs appears lower in the younger years of 0–29 months [55,61]. Animal food sources contain haem iron and retinol, which are both more bioavailable than the chemical forms of those nutrients found in plant foods. Furthermore, high cereal diets contain phytates which can additionally inhibit iron absorption [116]. Although iron and Vitamin A intake in younger children appear to be adequate, the bioavailability of these nutrients may be low. Additionally, the lower fat intake in children 0–3 years old, below the recommendation, could also reduce the availability of Vitamin A, as fat is needed to facilitate carotenoid absorption. The micronutrient deficiencies (Vitamins A and D, and selenium) and inadequacies (calcium; selenium; magnesium; Vitamins A, B1, B2, C, and D) in the diets of older children could be accounted for by low consumption of the following food groups: dairy, soybeans, seafood [93], nuts [108], fruits and vegetables [93,108], as well as diet diversity [42]. Vitamin D, calcium, and dairy are important nutrients and foods required for bone development in children. Infancy and childhood are important periods of life for bone development through the accrual of peak bone mass for skeletal maturity [117]. Poor bone health can have long-term adverse effects, such as osteoporosis in later life, which is of special concern in view of the ageing population of China [118]. The selenium content in soil is important to consider as a solution for selenium deficiency and intake inadequacies, as the selenium content of animal products, cereals, and plants can vary at least 10-fold based on the soil’s selenium content [116]. It is important to consider the regional differences in selenium status, as Hebei province was at risk of toxicity.

Globally, obesity and overnutrition appear to be of increasing nutritional concern especially among older children [6]. In this review, total fat, saturated fat, and sodium intake were all reported as above recommended limits. It is important to limit the intake of these nutrients due to their contribution to obesity and risk for other non-communicable diseases, such as hypertension. It is likely that energy was under-reported in this review, given the increasing overweight and obesity prevalence in Chinese children and the reported energy being below the EER. Under-reporting is a common phenomenon in dietary intake assessment methods, especially in overweight and obese participants [119].

Several other nutrients and foods are also at risk of being overconsumed. It was reported that in some areas, such as in Hebei province, excess iodine levels were found for up to 68% of the population [100]. Salt, being one of the most important condiments in the Chinese diet, was used as an iodine fortification vehicle, enacted via legislation with the universal salt iodization act (USI) in 1995. Iodine intake is therefore linked to sodium intake from salt, which was shown to be consumed at higher than recommended levels across all age groups. Thus, the level of iodine in the salt might need to be revisited, as it may result in excessive iodine intake, leading to iodine-induced thyroid dysfunction. Drinking water from shallow wells may also have high iodine content and may expose people to high levels; this was found in eastern China [120].

### 4.1. Strength and Limitations 

To the best of our knowledge, this is the first systematic review summarizing the available literature from the last 10 years on nutrient status, nutrient intake, food intake, and diet diversity in Chinese children 0 to 18 years of age. This review has several limitations, such as a lack of recent nutrient status and intake data at the national level. The latest national nutrient status data were collected in 2009 (not including anemia, which was last collected in 2014) for children 7 years and under and excluded calcium, folic acid, and Vitamin B12. National nutrient intake data for children 3 years and younger were missing, and thus we could only analyze sub-national or regional data. Additionally, national surveys, such as CHNS, do not cover all provinces of China. Sub-national studies, and in particular the data from baseline of intervention trials, which were included in the review, are in their nature limited in representativeness, containing lower subject numbers and frequently focusing on a specific group of interest. The prevalence of energy and nutrient adequacy was not always reported; therefore, the mean intake was assessed against several indicators, such as EAR, EAR, AI, %EI, and/or UL, depending on the reference values used in the individual study. These limitations do not allow accurate quantification of the inadequacy of intake. The adequacy of consumption of food groups and diet quality was even more difficult to assess, as the retrieved studies used a variety of scoring systems. A consistent approach to assessing the nutrient status and intake using the same food groups and scoring systems is warranted, especially in national studies.

### 4.2. Future Opportunities

Globally, there has been a shift in public health focus to move from nutrient to food-based approaches. This is because of the multiple social, economic, and health benefits associated with successful food-based approaches that lead to year-round availability of, access to, and consumption of nutritionally adequate amounts and varieties of foods [121]. Additionally, poor dietary choices leave gaps in micronutrient intake and nutritional deficiencies still exist [122]. Even though food fortification programs in China have been successful in reducing many nutritional issues in recent decades [68,105,123,124,125], it seems there are still pockets of nutritional deficits in specific age groups or regions that could require specific approaches. For example, a targeted food-based solution, focusing on specific food groups that were below recommended levels and nutrients that were deficient and/or inadequate, could be implemented. In older children, this could be dairy fortified with Vitamin A, Vitamin D, and/or selenium. For younger children, more bioavailable sources of Vitamin A and iron could be considered in terms of food fortification and supplementation. In addition, increasing dietary diversity can provide a more sustainable option to control malnutrition [126] and should be considered, especially in younger age groups, to develop lifelong healthy eating habits. Studies to assess the efficacy of these specific approaches need to be planned in the future. 

## 5. Conclusions

Overall, anemia and iron deficiency remain key issues among the youngest age groups, especially in the western rural population of China. VAD remains of high concern in all age groups among Chinese children. Additionally, in older children, deficiencies in Vitamin D and selenium are nutritional issues. Nutrient inadequacies (iron; calcium; selenium; zinc; and Vitamins A, B1, B2, and C intake), in parallel with excess intake of total and saturated fat and sodium, tended to increase with age. The consumption of dairy, soybean, nuts, seafood, fruit and vegetables, and diet diversity could be increased, especially in younger children. Given various nutrition issues across age groups, seasons, and regions of China, nutrition interventions should be tailored to specific groups and geographically relevant areas.

## Figures and Tables

**Table 2:** Summary table showing characteristics of national and subnational studies included in the review.

Name of Study/Description/Reference	Year of Study	Provinces ^1^	Age (yr)	Subject Number (n)	Outcome Indicator	Nutrients/Food
National studies						
Chinese Nutrition and Health Surveillance (CNHS) 2013; Duan et al. (2020) [25], Pang et al. (2021) [26]	2013	Not named: 30 provinces	2–4	14,850	Food intake (% consumed) Nutrient status	Dairy/milk, eggs, fruit Vitamin D
China Health and Nutrition Survey (CHNS) 2015; Jia et al. (2020) [27], Hu et al. (2021) [28], Zhao et al. (2021) [29]	2015	Beijing, Chongqing, Guangxi, Guizhou, Heilongjiang, Henan, Hubei, Hunan, Jiangsu, Liaoning, Shaanxi, Shandong, Shanghai, Yunnan, Zhejiang	3–17	1122–1699	Food intake (g/day) Nutrient intake	Dairy and milk Energy, multiple nutrients: protein, fat, carbohydrates, retinol, thiamine, riboflavin, Vitamin C, calcium, magnesium, selenium, zinc, iron, phosphorus, copper
Chinese Health Nutrition Survey (CHNS) 2010–2012 (Zhang et al. (2017), Younzi et al. (2018), Jinghuan et al. (2015), Li et al. (2017), Wang et al. (2017), Meng et al. (2018), Liu et al. (2017), Liu et al. (2016), Yang et al. (2015), Zhang et al. (2017), Zhang et al. (2017), Yang et al. (2017), Hu et al. (2017), Afeiche et al. (2018), Ding et al. (2022) [22,30,31,32,33,34,35,36,37,38,39,40,41,42,43]	2010–2012	Beijing, Chongqing, Guangxi, Guizhou, Heilongjiang, Henan, Hubei, Hunan, Jiangsu, Liaoning, Shaanxi, Shandong, Shanghai, Yunnan, Zhejiang	3–18	335–33,016	Nutrient status Nutrient intake Food intake (% consumed) Diet quality	Anemia, iron, Vitamin A, D, selenium, zinc, iodine status Energy, multiple nutrient intakes: protein; fat; added sugar; Vitamins A, B1, B2, B3, D, C; calcium; iron; zinc; selenium; phosphorus; magnesium; sodium Multiple food groups: meat, eggs, milk, poultry and fish, and their products DDS ^2^
National School-based obesity intervention trial; Gui et al. (2017) [44]	2013	Liaoning, Tianjin, Ningxia, Shanghai, Chongqing, Hunan, Guangdong	6–17	53,151	Food intake (g/% consumed)	Sugar-sweetened beverages
National IDD surveillance (Liang et al. (2017), Wang et al. (2017) [45,46]	2011	Beijing, Chongqing, Guangxi, Guizhou, Heilongjiang, Henan, Hubei, Hunan, Jiangsu, Liaoning, Shaanxi, Shandong, Shanghai, Yunnan, Zhejiang	8–10	15,003–130,000	Nutrient status	Iodine
Chinese National Survey on Students Constitution and Health (CNSSCH) 2014; Yang et al. (2020) [47]	2014	Not named: 30 provinces	9–14	48,537	Nutrient status Food intake (% consumed)	Anemia Eggs, milk
Chinese Environmental Exposure-Related Human Activity Patterns Survey (CEERHAPS-C); Guo et al. (2020) [48]	2013–2016	Not named: 30 provinces	6–17	41,439	Food intake (ml/month)	Milk
Subnational						
Nutrition Improvement Project on Children in Poor Areas of China (NIPCPAC); Feng et al. (2022) [49]	2018	Guizhou, Henan, Xinjiang, Hebei, and Hubei	0.5–2	1522	Nutrient status Food intake	Iron, calcium, zinc MDD, MMF, MAD
Maternal and health facility (Jia et al., 2015) [50]	2012	Beijing, Guangdong, Shanghai, Sichuan, Jiangsu	0.5–2	750	Nutrient status Nutrient intake	Anemia Multiple nutrient intakes: protein; Vit A, B1, B2, B3, C; calcium; iron
WFR, infants and young children, cross-sectional; Li et al. (2017) [51]	2014	Beijing, Guangdong, Hebei, Guangxi	0.5–2	472	Nutrient intake	Energy, multiple nutrient intakes: protein; Vit A, B1, B2, B3, B6, C; calcium; iron; zinc; selenium; phosphorus; magnesium
Rural infants and young children intervention; Huo et al. (2015) [52]	2010	Sichuan, Shaanxi, Gansu	0.5–2	1290	Nutrient status	Anemia
Wenchuan Earthquake, infants and young children; Sun et al. (2013) [53]	2010	Sichuan, Shaanxi, Gansu	0.5–2	1237	Nutrient status Diet quality	Anemia MDD ^3^, MMF ^4^, consumption of iron-rich foods
Rural infants and young children, cross-sectional; Wang et al. (2017) [54]	2011	Gansu, Qinghai, Xinjiang, Sichuan, Guizhou, Yunnan, Tibet	0.5–2	1938/3258	Food intake (% consumed) Diet quality	Multiple food groups: grains, roots and tubers; legumes and nuts; dairy; flesh foods; eggs; Vit A; F&V; other F&V MDD ^3^
Rural infants and toddlers, cross-sectional; Huang et al. (2019) [55]	2018	Qinghai	0.5–2	856	Nutrient status	Anemia, hemopoietic nutrients (iron, Vit B12 and A, and folic acid)
Rural infants and toddlers, complementary food supplements intervention; Zhang et al. (2016) [56]	2012	Qinghai	0.5–2	804	Nutrient status Diet quality	Anemia MDD ^3^, MMF ^4^
Rural infants and young children, complementary food supplements intervention; Dong et al. (2013) [57]	2010–2011	Gansu	0.5–2	314	Nutrient status	Anemia
Rural infants and young children, cross-sectional; Li et al. (2018) [58]	2014	Yunnan	0.5–2	938	Nutrient status	Vit D
Rural infants and young children, cross-sectional; (Yao et al., 2022) [59]	2018	Sichuan	0.5–2	1522	Food intake	MDD, MMF
Dietary pattern and anemia; Zou et al. (2021) [60]	2015 and 2018	Hunan	0.5–2	288	Anemia Food intake	MDD MMF MAD Consumption of iron-fortified food
Rural children, micronutrient powder intervention; Luo et al. (2017) [61]	2013	Shaanxi	0.5–2.5	610	Nutrient status	Anemia
Maternal and Infant Nutrition and Growth (MING) study; Li et al. (2017), Ma et al. (2014) [62,63]	2011–2012	Beijing, Guangdong, Shanghai, Sichuan, Gansu, Liaoning, Henan, Jiangsu	0.5–3	1078	Nutrient intake	Energy, multiple nutrients: energy; fat; carbohydrate; protein; fiber; Vitamins A, C, E, B1, B2, B3; calcium; phosphorus; magnesium; iron; zinc; sodium; selenium
Rural Mixed Methods Analysis Infants and Toddlers Study; Wang et al. (2018) [64]	2015–2017	Shaanxi, Hebei, Yunnan, Guizhou, Henan, Beijing	0.5–3	20,909	Nutrient status	Anemia
Rural early childhood development Intervention; Shi et al. (2020) [65], Zhao et al. (2021) [66]	2013	Guizhou, Shanxi	0.5–3	2236	Nutrient status Food intake	Anemia MMD, MMF, and MAD (Zhao et al.)
Rural infants and young children, Yingyanboa intervention (Wang et al. (2015), Wang et al. (2017) [67,68]	2010	Yunnan, Guizhou, Shaanxi	0–3	1370	Nutrient status Diet quality	Anemia; Fe; Vit A, D, B12; folic acid MDD ^3^
Vitamin A supplementation, Chongqing; Liu et al. (2021) [69]	2018	Chongqing	0–5	1016	Nutrient status	Vitamin A
Pre-school children, Health Examination Centers and Children Hospital; Zhang et al. (2020) [70]	2016	Chongqing	0–6	6953	Nutrient status	Vit D
Children, south China, cross-sectional; Guo et al. (2018) [71]	2016–2017	Guangdong	0–6	16,755	Nutrient status	Vit D
Children, association of Vit D with bone density; Fu et al. (2016) [72]	2014–2015	Jiangsu	0–7	4532	Nutrient status	Vit D
Outpatient children, hospital of Harbin; Wei et al. (2018) [73]	2014–2016	Heilongjiang	0–12	9795	Nutrient status	Vit D
Northeast Chinese Children, Serum cross-sectional; Chen et al. (2018) [74]	2017	Jilin	0–12	2096	Nutrient status	Vit A, D, E
Urban children, cross-sectional; Chang et al. (2014) [75]	2011	Sichuan	0.5–14	1218	Nutrient status	Anemia Vit D
Hospital-based cross-sectional study; 18 provinces; Yang et al. (2020) [76]	2016–2018	Inner Mongolia, Yunnan, Sichuan, Shaanxi, Guangxi , Beijing, Liaoning, Hebei, Shandong, Jiangsu, Zhejiang, Guangdong, Heilongjiang, Anhui, Hubei, Henan, Hunan	0–18	460,567	Nutrient status	Vitamin D
Semi-quantitative FFQ validation, young children, 24HR (3 days); Ma et al. (2022) [77]	2019	Shaanxi, Qinghai, Gansu	2–6	326	Nutrient intake	Energy and multiple nutrient intake: protein; fat; carbohydrate; fiber; Vitamins A, B1, B2, B3, C, D; iron; zinc; calcium; magnesium
12 cities central and western China; Chen et al. (2021) [78]	2019	Chongqing, Guizhou, Yunnan, Sichuan, Fujian, Shaanxi, Qinghai, Xinjiang	2–16	2194	Nutrient status	Vitamin A
Rural children, toxic metals, cross-sectional; Tian et al. (2020) [79]	2013	Hebei	3–5	80	Nutrient status Nutrient intake	Multiple nutrient statuses: calcium, iron, zinc, magnesium, selenium (urine) Multiple nutrient intakes: calcium, iron, zinc, magnesium, selenium
Urban pre-school children, Vitamin A supplement intervention; Chen et al. (2014) [80]	2011	Sichuan	3–6	500	Nutrient status	Anemia, iron, Vitamin A
Pre-school children, cross-sectional; Peng et al. (2014) [81]	2012	Chongqing	3–6	492	Nutrient status	Vit A
Nutrition Study of Pre-school Children and School Child; Ma et al. (2014), Zhao et al. (2017), Xue et al. (2015), Liu et al. (2018) [82,83,84,85]	2011–2012	Beijing, Guangdong, Sichuan, Liaoning, Gansu, Henan, Jiangsu, Hebei	3–12	423–1866	Nutrient status Nutrient intake Food intake (g/day) Diet quality	Anemia, Vit D Energy, multiple nutrient intakes: protein; fat; carbohydrate; fiber; Vitamins A, B1, B2, B3, C, D; iron; zinc Food groups: cereals, vegetables, fruits, meats, fish, eggs, milk, nuts, beans DDS ^5^ and SDDS ^6^
Rural vs. urban, children and adolescents, universal salt iodization policy; Zou et al. (2014) [86]	2014	Zhejiang	3–18	1970	Nutrient status	Iodine
Rural school-children, food insecurity, cross-sectional; Shen et al. (2015) [87]	2011	Guangxi, Yunnan	6–14	1583	Nutrient status	Anemia
China Child and Adolescent Cardiovascular Health (CCACH) 2012–2015; Yan et al. (2019), Li et al. (2020), Xiao et al. (2020) [88,89,90]	2012–2015	Beijing, Jilin, Shandong, Ningxia, Shanghai, Chongqing, Sichuan	6–18	10,696–12,618	Diet quality Nutrient status	Healthy Diet Score ^7^ Vit D
School children, northwest Chinese City, cross-sectional; Li et al. (2018) [91]	2015	Ningxia	6–18	1582	Nutrient status	Vit D
Rural, egg and milk supplementation; Zhao et al. (2021) [92]	2013–2015	Guangxi	7–8	818	Nutrient intake	Energy, protein, fat, iron, calcium, Vitamin B2
The Dietary Quality During Childhood (DQDC), cross-sectional; Cheng et al. (2016), Zhang et al. (2019) [93,94]	2013	Sichuan	7–14	1719–2043	Nutrient intake Food intake (g/day) Diet quality	Nutrient intakes: Vitamin A, fiber Multiple food intakes: grains, vegetables, fruits, dairy and products, soybeans and products, meat, fish and shrimp, eggs, SSB DDS ^8^
Chinese Nutrition and Health Surveillance 206-17, cross-sectional; Tian et al. (2022) [95]	2016–2017	Jiangsu	7–17	335	Nutrient status	Multiple nutrients: Vitamin A and D and zinc
Rural, FFQ, cross-sectional; Zhou et al. (2015) [96]	2012–2013	Jiangsu	7–17	516	Nutrient intake	Multiple
Rural vs. urban, children and adolescent, sub-set from CHNS 2011–2013; Zou et al. (2016) [97]	2011–2012	Zhejiang	7–17	1534	Nutrient status Nutrient intake	Anemia, iron status Energy, multiple nutrient intakes: protein; fat; carbohydrate; Vitamins A, B1, B2, B3, C; calcium; sodium; magnesium; iron; zinc; selenium
Rural vs. urban, sub-set from CHNS 2011–2013; Zou et al. (2021) [98]	2016–2017	Zhejiang	6–12	2818	Nutrient status	Vitamins A and D
Children and adolescents, sub-set of the national School Obesity Intervention; Tang et al. (2020) [99]	2013	Guangdong	7–18	2680	Nutrient status	Vit D
School-children, cross-sectional survey; Lv et al. (2012) [100]	2010	Hebei	8–10	1200	Nutrient status	Iodine
School-age children, cohort study; Wang et al. (2018) [101]	2012	Zhejiang	8–10	784	Nutrient status	Iodine
Rural children, eggs vs. chewable vitamins intervention; Weiner et al. (2013) [102]	2010	Gansu	9–12	2000	Nutrient status	Anemia
Rural school-children, school feeding program intervention; Wang et al. (2020) [103]	2012	Qinghai, Gansu, Shaanxi, Ningxia	10–12	8411	Nutrient status	Anemia
Rural school children, cross-sectional study; Tao et al. (2022) [104]	2018	Anhui , Yunnan, Henan	10–13	8690	Diet quality Food intake	DDS Multiple food groups: grains, tubers, vegetables, fruits, meat, fish, beans, nuts and seeds, dairy
Rural school-children, eggs vs. vitamin A supplement intervention; Cao et al. (2013) [105]	2010–2011	Chongqing	10–18	288	Nutrient status	Anemia Vit A
Iodine, 3 provinces; Shan et al. (2021) [106]	2016	Hebei, Guangxi, Zhejiang	11–12	3808	Nutrient status	Iodine, Vitamin A, Vitamin D
School-children, micronutrient-fortified milk intervention; Wang et al. (2017) [107]	2015	Shaanxi	12–14	137	Nutrient status	Multiple nutrients: iron; Vit D, B2, B12; selenium
Semi-quantitative FFQ validation, adolescents ; Liu et al. (2019) [108]	2018	Beijing, Chongqing	12–17	160	Nutrient intake Food intake (g/day)	Multiple food groups (cereal, tubers, fried cereal food, beans, fresh vegetables, salted vegetables, the fungus algae, fresh fruits, dairy, red meat, poultry, seafood, eggs, nuts, desserts, sugar beverages, tea/coffee)

^1^ Province region is indicated by colors; east China = yellow, central China = orange, western China = brown (UNICEF, 2022) [109]. ^2^ Defined by 9 food groups according to 2016 Chinese Dietary Guideline. ^3^ MDD = minimum diet diversity. ^4^ MMF = minimum meal frequency. ^5^ Defined by consumption of at least 10 gs of 10 food groups. ^6^ Defined by consumption of at least 10 gs of 17 food groups. ^7^ The Healthy Diet Score included the following 5 components: fruits and vegetables ≥1/d; fish or fish products ≥2/wk; milk, bean, or dairy products ≥1/d; fried food/Western fast food ≤2/wk; sugar-sweetened beverages ≤2/wk. ^8^ The Chinese Children Dietary Index: 16 components, which incorporated nutrients, foods/food groups, and health-promoting behaviors.

**Table 3:** The mean energy and fat intake range, reported from various studies across different age groups, compared with the estimate energy requirement (EER) kcal/day and %EI.

Age (yrs)	Energy Intake		Fat Intake		
	EER kcal/day (Female/Male)	Mean Intake (kcal/day)	Recommended % EI	Mean Intake (g/day)	%EI
0	549 ^b^	71–103 Ma et al. (2014) [63]	48 (AI)	24.7–28.3 Ma et al. (2014) [63]	18–21 ^b,c^
0.5	684 ^b^	744 Li et al. (2017) [62]	40 (AI)	25.7 Li et al. (2017) [62]	15 ^b,c^
1	900/800	1146 Li et al. (2017) [62]	35(AI)	39.4–44.6 ^+^ Li et al. (2017) [62], Ma et al. (2022) [77]	18–19 ^b,c^
2	1100/1000	1184–1523 ^+^ (Li et al., 2017) [62], Ma et al. (2022) [77]	20–30		
3	1250/1200	1523 ^+^–1553 Ma et al. (2022) [77], Meng et al. (2018) [42]	20–30		
4	1300/1250	1299–1628 ^a^ Zhang et al. (2017) [38], Meng et al. (2018) [42], Xue et al. (2015) [84], Ma et al. (2022) [77]	20–30	44.6 ^+^ Ma et al. (2022) [77]	37 ^a^ Zhang et al. (2017) [38]
5	1400/1300	1299–1628 ^a^ Zhang et al. (2017) [38], Zhao et al. (2021) [29], Meng et al. (2018) [42], Xue et al. (2015) [84], Ma et al. (2022) [77]	20–30		
6	1600/1450	1299–1672 ^a^ Zhang et al. (2017) [38], Zhao et al. (2021) [29], Meng et al. (2018) [42], Xue et al. (2015) [84], Ma et al. (2022) [77]	20–30		
7	1700/1550	1416–1672 ^a^ Zhang et al. (2017) [38], Zhao et al. (2021) [29], Meng et al. (2018) [42]	20–30	52.6–66.2 ^a^ Zhang et al. (2017) [38], Zhao et al. (2021) [29]	37–38 ^a^ Zou et al. (2016) [97], Zhang et al. (2017) [38], Zhao et al. (2021) [29]
8	1850/1700	1416–1672 ^a^ Zhang et al. (2017) [38], Zhao et al. (2021) [29], Meng et al. (2018) [42]	20–30	30.5–39.4 Zhang et al. (2017) [38], Zhao et al. (2021) [29]	
9	2000/1800	1416–1672 ^a^ Zhang et al. (2017) [38], Zhao et al. (2021) [29]	20–30		
10	2050/1900	1416–1672a Zhang et al. (2017) [38], Zhao et al. (2021) [29], Meng et al. (2018) [42]	20–30		
11	2350/2050	1417–2050 Zou et al. (2016) [97], Lui et al. (2019) [108], Zhang et al. (2017) [38] Zhao et al. (2021) [29], Yunzi et al. (2018) [39], Meng et al. (2018) [42]	20–30	52.6–66.2 a	36–37 ^a,c^ Zou et al. (2016) [97], Lui et al. (2019) [108], Zhang et al. (2017) [38], Zhao et al. (2021) [29]
14–17	2850/2300	1416–2050 Zou et al. (2016) [97], Lui et al. (2019) [108], Zhang et al. (2017) [38], Zhao et al. (2021) [29], Yunzi et al. (2018) [39], Meng et al. (2018) [42]	20–30	52.6–66.2 ^a^	32–37 ^a,c^ Zou et al. (2016) [97], Lui et al. (2019) [108], Zhang et al. (2017) [38] Zhao et al. (2021) [29]

^a^ National data; Zhang et al. (2017) [38], Zhao et al. (2021) [29], Meng et al. (2018) [42]. ^b^ Calculated EER for energy based on recommendations of 80–90 kcal/kg/day, using 6.1 kg and 8.55 kg, respectively, for 0–0.4 years old and 0.5–0.9 years old. ^c^ Calculated based on g/day, ^+^ median. Green = above EER or %EI; gray = above EER or %EI for national studies, but inadequacies exist in smaller studies; Orange = below EER or %EI.

**Table 4:** Anemia prevalence and mineral status (% deficiency), intake, and inadequacy per age group. The mean nutrient intake range has been reported as % inadequacy across different age groups compared with the EAR.

Nutrient	Age (Years)	0–0.4	0.5–0.9	1–3.9	4–6.9	7–10.9	11–13.9	14–17
Anemia	% prevalence	46	17–86	4–30	5.5 ^a^–21	4.3 ^ab^–21	7.2 ^ab^–21	7.2 ^ab^–21
Iron	% deficiency	-	48–62	10–62	10–38	8–18	7.3–9.4	7.3–8.3
	Mean intake (mg/day)	3.1–4.8	6.6–8.7	8.8–23.7 ^+^	11.2 ^a^–23.7 ^+^	14.0 ^a^–30.4	17.4 ^a^–30.4	19.5 ^a^–30.4
	% inadequacy				9.7 ^a^	18 ^a^	24 ^a^	19 ^a^
Zinc	% deficiency					4–10 ^a^	4	4
	Mean intake (mg/day)	3.1–4.5	4.0–4.8	4.8–7.7	6.3 ^ab^–9.3	7.7 ^a^–12.5	8.5 ^ab^–12.5	8.5 ^ab^–12.5
	% inadequacy	0.4–19			24 ^a^	28 ^a^–56	30 ^a^–56	30 ^a^–56
Selenium	% deficiency					44 ^a^	3.1–3.6	
	Mean intake (mg/day)		15.4–18.6 ^+^	27 ^ab^–31.1	33.8 ^a^–70.0	32.2 ^a^–33.8 ^a^	33.8 ^a^–38.1 ^a^	36 ^a^–45.6 ^a^
	% inadequacy				51 ^a^	53 ^a^–64 ^a^	71 ^a^	73 ^a^
Calcium	Mean intake (mg/day)	322–487	355–577	413–801 ^a^	236 ^a^–642	273 ^a^–741	306 ^a^–741	306 ^a^–741
	% inadequacy				97 ^a^	98 ^a^	99 ^a^	97 ^a^
Magnesium	Mean intake (mg/day)	-	112	153–369 ^+^	203 ^a^–369 ^+^	203 ^a^–214 ^a^	203 ^a^–278 ^a^	214 ^a^–278 ^a^
Sodium	Mean intake (mg/day)	57–76	518	571 ^+^–2470	3148 ^ab^–4370	3787 ^a^–3360 ^ab^	4228 ^a^–4400	3360–4849 ^a^

**^a^** National data (Jinghuan et al. (2019), Li et al. (2017), Yang et al. (2020), Wang et al. (2017), Liu et (2016), Hu et al., 2021) [22,28,30,31,36,47]; ^b^ sub-national data omitted, in preference of national data. Green = above EAR/RNI (all except sodium); gray = above EAR for national studies, but inadequacies exist in smaller studies; Orange = below EAR (all except sodium). Sodium: green = below AI and orange = above AI and/or PI = Chinese proposed intake for preventing non-communicable chronic diseases (PI-NCD) ^+^ median.

**Table 5:** Vitamin deficiency (%), intake range (units per day), and % inadequacy reported for Vitamins D, A, B1, B2, B3, and C across different age groups compared with EAR and UL.

Nutrient	Age (Years)	0–0.4 ^b^	0.5–0.9 ^b^	1–3.9	4–6.9	7–10.9	11–13.9	14–17
Vit A	% deficiency % VAI	21–88 11.2–43	20–21 40–58	0–21 43–54	8.9–20 39–44	6.6 ^ac^–27 ^a^ 17 ^ac^–38	0.9–27 ^a^ 15	4.5–27 ^a^ 14 ^a^–21 ^a^
	Mean intake (µg/day)	620–709	455–670	432–867	482–522 ^a^	227–624 ^a^	223–661 ^a^	227–769 ^a^
	% inadequacy	0.4–24			25 ^a^	31 ^a^–70 ^a^	42 ^a^–70 ^a^	3–70 ^a^
Vit B1	Mean intake (mg/day)	0.2–0.5	0.3–0.5	0.3–0.8 ^+^	0.5 ^a^–0.8	0.9–0.7 ^a^	0.6–0.7 ^a^	0.8 ^a^–1.2
	% inadequacy				68 ^a^	75 ^a^–79 ^a^	75 ^a^–89 ^a^	75 ^a^–92 ^a^
Vit B2	Mean intake (mg/day)	0.6–0.9	0.8–1.1	1.0–1.4 ^+^	0.5 ^a^–1.4 ^+^	0.5–0.6 ^a^	0.67 ^a^–1.0	0.67 ^a^–1.0
	% inadequacy				66 ^a^	77 ^a^	84 ^a^	86 ^a^
Vit B3	Mean intake (mg/day)	2.1–2.9	4.2–4.6	5.8–16 ^+^	11–16 ^+^	9.5–10 ^a^	9.5–10 ^a^	9.5–15 ^a^
	% inadequacy							
Vit C	Mean intake (mg/day)	58–76	45–54	59–88	43 ^a^–75 ^+^	28–61 ^a^	25–64 ^a^	25–66 ^a^
	% inadequacy				54 ^a^	58 ^a^–65 ^a^	65 ^a^–69 ^a^	65 ^a^–76 ^a^
Vit D	% deficiency	0–28	0–28	0–34	1–58	1.8–41 ^a^	1.8–50 ^a^	1.8–52 ^a^

Blue = above UL; green = above EAR; light green = above EAR for national studies, but inadequacies exist in smaller studies; gray = above and below EAR (inconsistencies exist between studies); orange = below EAR; ^a^ national data (Yang et al. (2015), Zhang et al. (2017), Zhang et al. (2017), Wang et al. (2017), Meng et al. (2018), Hu et al. (2017), Hu et al. (2021)) [28,32,34,36,40,41,42,98]; ^b^ compared with Adequate Intake (AI); ^c^ sub-national data omitted; ^+^ median.

**Table 6:** Food group consumption of Chinese children (between 3 and 17 years) across general, highly urban, moderately urban, and rural areas. Recommendations are based on the 2016 Chinese Food Pagoda for general population of healthy people over 2 years of age.

Population Group (Years)	Measure	Milk and Milk Products	Beans, Nuts, and Seeds	Red Meat	Poultry	Seafood	Eggs	Fruits	Vegetables	Cereals, Tubers, and Legumes
	Recommended g/day	300	25–35	40–75	40–75	40–75	40–50	200–350	300–500	250–400
General (3–4) ^a^ [27] (5–8) ^a^ [27] (7–15) ^b^ [108] (7–17) ^e^ [93]	g/day (mean)	61	-							
		41	-							
		230	28 ^c^	113	24	0	20	96	127	277 ^d^
		200	-	129	-	13	34	142	154	550
(3–4) ^a^ [27] (5–8) ^a^ [27]	% below recommended	96–99	-	-	-	-	-	-	-	-
Highly urban (4–17) ^a^ [38]	g/day mean (SE)	120 (4)	-	223 (9)	74 (4)	40 (2)	65 (2)	-	-	-
Moderately urban (4–17) ^a^ [38]	g/day mean (SE)	116 (14)	-	200 (0.4)	72 (4)	38 (2)	51 (2)	-	-	-
Rural (4–17) ^a^ [38]	g/day mean (SE)	99 (9)	-	181 (5)	71 (4)	33 (1)	50 (1)	-	-	-

Orange = below recommended, green = within recommended, blue = above recommended. ^a^ National data (Jia et al. (2020) [27], Zhang et al. (2017) [38]). ^b^ Beijing, Chongqing (Liu et al. (2019) sub-national study) [108]. ^c^ Calculated based on 5 g/day of soybeans, 21.5 g/day of beans, and 1.9 g/day of nuts (Cheng et al. (2016) sub-national study) [93]. ^d^ Grains = 217 g, tubers = 37 g, fried cereals = 23 g; children 12–17 years. ^e^ Chengdu (Cheng et al. (2016); sub-national study) [93].

## Data Availability

The complete dataset for this review is presented across the tables and Appendix A and Appendix B.

## Supplementary Materials

The following supporting information can be downloaded at: https://www.mdpi.com/article/10.3390/nu15061536/s1, Table S1: The mean carbohydrate intake range, reported from different studies across different age groups compared with EAR, %EI; Table S2: The mean protein intake range, reported from different studies across different age groups compared with RNI, EAR, and UL; Table S3: The mean phosphorus intake range, reported from different studies across different age groups compared with RNI, EAR, and UL.

## Appendix A

**Table A1:** Keywords and PICOS criteria to define the research question.

Keywords	(‘diet’ OR ‘nutrient’ OR ‘micronutrient’ OR ‘vitamin’ OR ‘mineral’)	AND
	(‘malnutrition’ OR ‘intake’ OR deficiency’ OR ‘inadequate’ OR ‘anaemia’)	AND
	(‘infant’ OR ‘toddler’ OR ‘preschool’ OR ‘child’ OR ‘adolescent’)	AND
	(‘China’ OR ‘Chinese’)	AND
	(‘pregnant’ OR ‘maternal’)	NOT
Parameter	Description	
Population	Healthy children (0–18 years old) living in China	
Intervention	Not applicable	
Comparison	Age-specific WHO/national cutoffs; regions in China	
Outcomes	Nutrient deficiencies via biochemical or inadequacies via dietary intake studies. Food intake/diet diversity via dietary intake studies.	
Setting/Design	Observational studies (i.e., cross-sectional, cohort, case–control and longitudinal studies, case reports), intervention baseline results.	

## Appendix B

**Table A2:** Diet quality measures and results of Chinese children (0.5–17 years).

Diet Quality Measure	Study	Based on	Cutoff	Result	Population Group (Years; Region)
Minimum dietary diversity (MDD), minimum meal frequency (MMF), and minimum acceptable diet (MAD)	Zhao et al. (2020) [65] based on dedicated cross-sectional survey	WHO guidelines for measurement of infant and young child feeding; 1-time 24 h dietary recall	MMF, MMD, and MAD according to WHO and UNICEF, 2017	60.8% of children met MMF. 23.0% met MDD; 17.6% met MAD; Dietary Frequency: roots and tubers 80.1%; fruits 69.2%; meat 58.7%; dairy 47.2%; legumes and nuts 24.7%; egg 22.3%; Vitamin-A-rich foods 62%; 54% were breastfed	6–23 months; from 6 rural counties in Shanxi and Guizhou provinces
Minimum dietary diversity (MDD), minimum meal frequency (MMF), and minimum acceptable diet (MAD)	Feng et al. (2022) [55]; data from the Nutrition Improvement Project on Children in Poor Areas of China (NIPCPAC) in 2018	24 h dietary recall	MMF, MMD, and MAD according to WHO and UNICEF, 2017	% of children meeting indicators: MDD = 68.9%, MMF = 77.9%, and MAD = 46.4%	6–23 months in rural communities in Guizhou, Henan, Xinjiang, Hubei, and Hebei provinces in 2018
Continued breastfeeding; minimum dietary diversity (MDD); consumption of iron-rich foods; anemia	Yao et al. (2021) [59] baseline data from a community-based child health counselling intervention	24 h dietary recall	Continued breastfeeding rate at 1 year, MMD, anemia	Continued breastfeeding rate at 1 year = 69.5%; MDD 49.5%; consumption of iron-rich foods = 55%; anemia: 52.1%	6–23 months, poor rural areas in Liangshan in 2018
Diet Diversity Score (DDS)	Meng et al. (2018) [38], using CHNS data (over a 3 day period)	9 food groups from the Chinese Dietary Guideline	DDS score lower than 8 was considered below the recommendation	6.14	3–12 years; National
				6.11	13–17 years; National
DDS	One 24 h, Zhao et al. (2017) [78]	10 food groups based on FAO; based on 10 g or more of food	No cutoff	5.60	6–12 years
17-food-group dietary diversity scores (SDDS)	One 24 h, Zhao et al. (2017) [78]	17 food groups; based on 10 g or more of food	No cutoff	7.5	6–12 years
Overall Food Variety Score (OFVS)	One 24 h, Zhao et al. (2017) [78]	Number of food items in 24 h. Single foods defined based on China Food composition table	No cutoff	13.2	6–12 years
Healthy Diet Score	The Chinese Child and Adolescent Cardiovascular Health (CCACH) (Yan et al. (2019) [83]	This is defined by the American Heart Association to determine cardiovascular health status.	Daily fruit and vegetable consumption, fish or fish product consumption ≥ 2 times per week, daily bean or dairy products, fried or Western fast food ≤ 2 times per week, and sugar-sweetened beverages (SSB) ≤ 2 times per week.	8.7% met the ‘Healthy Diet’ Score; the study reported that 43% of children met the goal for fruits and vegetables, 22% for seafood, 18% for beans and dairy, 60% for SSB, and 84.6% for fried/Western foods	6–17 years
The Chinese Children Dietary Index	Cheng et al. (2016) [87], Zhang et al. (2019) [88]	16 components with a maximum score of 160 points	No cutoff	88	7–15 years old; Chengdu, Sichuan, western China
Minimum acceptable diet (MAD)	Last 24 h before the study, WHO indicators on Infant and Young Child Feeding, Zhang et al. (2016) [56]	Combination of minimum dietary diversity and minimum meal frequency	No cutoff	MAD was not met 93–95%	6–23 months; rural Qinghai province; Guinan county and Huzhu county
% meeting dietary China Dietary Guidelines Index for Youth (CDGI-Y)	Ding et al. (2022) [43] using China National Nutrition and Health Survey (CNNHS) 2010–2012	Food frequency questionnaire: the CDGI-Y is composed of 12 food groups and 2 nutrient-related components: energy balance and energy supply from carbohydrate	CDGI-Y	20% of children met the guideline (21.9% girls and 18.2% boys) CDGI-Y score = 62.6 ± 11.0 (63.4 ± 10.9 girls and 61.8 ± 11.0 boys)	9–15 years; National 2010–2012
Dietary Diversity (DDS) and Dietary Frequency (DFS)	Tao et al. (2020) [98] using dedicated surveys in 90 rural primary schools	24 h dietary recall; 9 food groups based on FAO guidelines	No cutoff	Overall DDS = 5.56. DFS: grains = 1.34; vegetables = 1.23; meat = 1.16; fruits = 1.03; tubers: 0.99; dairy: 0.98; beans and nuts = 0.96; eggs: 0.95; fish: 0.71	10–13 years; rural; 3 provinces in China in 2018
