# Supplementary material for: Systematic Literature Review of the Nutrient Status, Intake, and Diet Quality of Chinese Children across Different Age Groups
Source: Nutrients. 2023 Mar 22;15(6):1536. doi: 10.3390/nu15061536 (PMC10057142; doi:10.3390/nu15061536)
Supplement: Supplementary file 1 [file nutrients-15-01536-s001.zip › nutrients-2181447-supplementary.pdf]

Table S1. The mean carbohydrate intake range, reported from different studies across different age groups compared to EAR, %EI. Green= above and within %EI.

Age	EAR g/day	%E	Mean intake (g/day)	%EI	Ref
0	65 (AI)	-	57.7- 75.3		[1]
0.5	80 (AI)	-	105		[2]
1	120	50-65	154.8- 243+		[2] [3]
4	120	50-65	225-243+		[4] [3]
7	150	50-65	182.42		[5]
11	150	50-65	182.42-282.2g/day	59%[a]	[6][5] [7]
14	150	50-65	182.42-282.2g/day	59%[a]	[6][5] [7]

[a] National data [+] median

Table S2. The mean protein intake range, reported from different studies across different age groups compared to RNI, EAR and UL. Green= above EAR/RNI.

Age	RNI	EAR	UL	Mean intake (g/day)	Ref
0	9 (AI)	-	-	11-14	[1]
0.5	20	15	-	18.3-24.2	[8][9] [2]
1	25	20	-	26.3-79[+]	[8][9][9] [3]
4	30	20	-	55.77-79[+]	[4]
7	30	25	-	48.3-116	[10] [5] [3]
11	30	25	-	48.3-116	[10] [5]
14	35	25	-	48.3-116	[10] [5] [7]

[+] median

Table S3. The mean phosphorus intake range, reported from different studies across different age groups compared to RNI, EAR and UL. green= above EAR/RNI.

Age	RNI	EAR	UL	Mean intake (mg/day)	% inadequacy	Ref
0	100 (AI)	-	-	158-278		-
0.5	180 (AI)	-	-	441		[2]
1	300	250	-	760-942		[2] [3]
4	350	290	-	698[a]-942[a]	10% [a,c]	[11] [3]
7	470	400	-	690-698[a]		[11] [5]
11	640	540	-	698[a]-1106	13% [a,d]	[11][7][5][b]
14	710	590	-	935[a]-1106		[11] [7]

[a] National data [b]= 690 omitted due to presence of national data [c] age group 3-12 years old [d] age group 13-17 years old.

References

1. Ma, D.; Ning, Y.; Gao, H.; Li, W.; Wang, J.; Zheng, Y.; Zhang, Y.; Wang, P. Nutritional Status of Breast-Fed and Non-Exclusively Breast-Fed Infants from Birth to Age 5 Months in 8 Chinese Cities. *Asia Pac J Clin Nutr* **2014**, *23*, 282–292, doi:10.6133/apjcn.2014.23.2.16.

2. Li, Z.; van der Horst, K.; Edelson-Fries, L.R.; Yu, K.; You, L.; Zhang, Y.; Vinyes-Pares, G.; Wang, P.; Ma, D.; Yang, X.; et al. Perceptions of Food Intake and Weight Status among Parents of Picky Eating Infants and Toddlers in China: A Cross-Sectional Study. *Appetite* **2017**, *108*, 456–463, doi:10.1016/j.appet.2016.11.009.

3. Ma, Y.; Tan, J.; Tan, Z.; Shang, L. Validity and Reliability of Semiquantitative Food Frequency Questionnaires for Assessing Nutrient Intake among Preschool Children

in Northwest China. *Hindawi Journal of Healthcare Engineering* **2022**, doi:10.1155/2022/1677252.

4. Xue, Y.; Zhao, A.; Cai, L.; Yang, B.; Szeto, I.M.Y.; Ma, D.; Zhang, Y.; Wang, P. Growth and Development in Chinese Pre-Schoolers with Picky Eating Behaviour: A Cross-Sectional Study. *PLoS One* **2015**, *10*, doi:10.1371/journal.pone.0123664.

5. Zou, Y.; Zhang, R.-H.; Xia, S.-C.; Huang, L.-C.; Fang, Y.-Q.; Meng, J.; Chen, J.; Zhang, H.-X.; Zhou, B.; Ding, G.-Q. The Rural-Urban Difference in BMI and Anemia among Children and Adolescents. *Int J Environ Res Public Health* **2016**, *13*, doi:10.3390/ijerph13101020.

6. Yunzi, A.; Yu, L.; López-Olmedo, N.; Popkin, B.M.; Pac, A.; Clin, J.; Author, N. Analysis of Dietary Trends in Chinese Adolescents from 1991 to 2011. *Asia Pac J Clin Nutr* **2018**, *27*, 1106–1119, doi:10.6133/apjcn.042018.02.

7. Liu, D.; Ju, L.; Yang, Z.; Zhang, Q.; Gao, J.; Gong, D.; Guo, D.; Luo, S.; Zhao, W. Food Frequency Questionnaire for Chinese Children Aged 12-17 Years: Validity and Reliability. *Biomedical and Environmental Sciences* **2019**, *32*, 486–495, doi:10.3967/bes2019.066.

8. Jia, N.N.; Zhang, S.; Li, T.; Tan, Z.; Yin, Y.; Chen, L.; Li, C.; Liu, A.; Bindels, J.G.J.G.; Dai, Y. Dietary Survey of Anaemic Infants and Young Children in Urban Areas of China: A Cross-Sectional Study. *Asia Pac J Clin Nutr* **2015**, *24*, 659–664, doi:10.6133/apjcn.2015.24.4.05.

9. Li, H.Z.; Jia, H.X.; Liang, D.; Deng, T.T.; Niu, L.T.; Han, J.H. [Study on the Contribution Rate of Follow-up Formula to the Nutrient Intake of Infants and Young Children Aged 7-24 Months in China]. *Zhonghua Yu Fang Yi Xue Za Zhi* **2017**, *51*, 65–69, doi:10.3760/cma.j.issn.0253-9624.2017.01.013.

10. Zhou, H.; Wang, S.; Yin, H.; Chen, C.; Yang, Y.; Jiang, M.; Yang, L.; Guo, B.; Tang, H.; Zhang, Y.; et al. Nutrition and Health Survey of Children and Youth in Lishui District, Nanjing City. *Wei Sheng Yan Jiu* **2015**, *44*.

11. Meng, L.; Wang, Y.; Li, T.; Loo-Bouwman, C.A. van; Zhang, Y.; Man-Yau Szeto, I.; Annika Van Loo-Bouwman, C.; Zhang, Y.; Szeto, I.M.-Y.I.M.-Y.I.M.Y.; van Loo-Bouwman, C.A.C.A.; et al. Dietary Diversity and Food Variety in Chinese Children Aged 3–17 Years: Are They Negatively Associated with Dietary Micronutrient Inadequacy? *Nutrients* **2018**, *10*, 1674, doi:10.3390/nu10111674.
